# A Luciferase-Expressing *Leishmania braziliensis* Line That Leads to Sustained Skin Lesions in BALB/c Mice and Allows Monitoring of Miltefosine Treatment Outcome

**DOI:** 10.1371/journal.pntd.0004660

**Published:** 2016-05-04

**Authors:** Adriano C. Coelho, Jordana C. Oliveira, Caroline R. Espada, Juliana Q. Reimão, Cristiana T. Trinconi, Silvia R. B. Uliana

**Affiliations:** Departamento de Parasitologia, Instituto de Ciências Biomédicas, Universidade de São Paulo, São Paulo, Brazil; Institut Pasteur, FRANCE

## Abstract

**Background:**

*Leishmania braziliensis* is the most prevalent species isolated from patients displaying cutaneous and muco-cutaneous leishmaniasis in South America. However, there are difficulties for studying *L*. *braziliensis* pathogenesis or response to chemotherapy *in vivo* due to the natural resistance of most mouse strains to infection with these parasites. The aim of this work was to develop an experimental set up that could be used to assess drug efficacy against *L*. *braziliensis*. The model was tested using miltefosine.

**Methodology/Principal Findings:**

A *L*. *braziliensis* line, originally isolated from a cutaneous leishmaniasis patient, was passaged repeatedly in laboratory rodents and further genetically manipulated to express luciferase. Once collected from a culture of parasites freshly transformed from amastigotes, 10^6^ wild type or luciferase-expressing stationary phase promastigotes were inoculated subcutaneously in young BALB/c mice or golden hamsters. In both groups, sustained cutaneous lesions developed at the site of inoculation, no spontaneous self- healing being observed 4 months post-inoculation, if left untreated. Compared to the wild type line features, no difference was noted for the luciferase-transgenic line. Infected animals were treated with 5 or 15 mg/kg/day miltefosine orally for 15 days. At the end of treatment, lesions had regressed and parasites were not detected. However, relapses were observed in animals treated with both doses of miltefosine.

**Conclusions/Significance:**

Here we described experimental settings for a late-healing model of cutaneous leishmaniasis upon inoculation of a luciferase-expressing *L*. *braziliensis* line that can be applied to drug development projects. These settings allowed the monitoring of the transient efficacy of a short-term miltefosine administration.

## Author Summary

*Leishmania braziliensis* is the most prevalent species isolated from patients displaying either cutaneous or mucocutaneous leishmaniasis in South America. In this study, we developed a transgenic luciferase-expressing *L*. *braziliensis* line. These parasites were passaged in hamsters and mice and then transformed back into promastigotes. Once inoculated subcutaneously in the footpad of young laboratory animals—BALB/c mice or golden hamsters, rapid and sustained footpad thickness increase developed. This experimental model was used to monitor the parasite load fluctuations and the response to miltefosine treatment. Mice were treated orally over a two-week period, starting at week 4 post-inoculation. Though such a regimen was shown to display efficacy, the effect was not sustained and both parasite re-expansion and delayed footpad thickness increase were noticed.

## Introduction

*Leishmania* spp. are the etiological agents of leishmaniasis, a complex of vector-borne infectious diseases transmitted by sand flies. The disease is widespread in the world in tropical and subtropical regions. Approximately 12 million people are affected worldwide and 1.2 million new cases occur each year [1]. Leishmaniasis is responsible for a spectrum of clinical manifestations including visceral, cutaneous, mucosal, disseminated and diffuse cutaneous disease that are determined, at least partially, by the species of the parasite [2]. In South America, *Leishmania (Viannia) braziliensis* is the most prevalent species isolated from patients displaying either cutaneous or mucocutaneous leishmaniasis [3–5]. This species is also associated with disseminated leishmaniasis [5].

In spite of its epidemiological importance, the study of *L*. *braziliensis* infections is limited by the difficulty of establishing good animal models of disease. *L*. *braziliensis* infects hamsters and induces a chronic cutaneous disease with no spontaneous healing [6, 7]. However, inbred strains of mice are generally resistant to *L*. *braziliensis* or develop a transient and self-healing cutaneous lesion [7–10]. When the parasites are inoculated into partially susceptible strains such as BALB/c mice, a non-ulcerative lesion develops initially and evolves to spontaneous healing in about 30 days [8, 10]. A mouse model of sustained skin lesions with *L*. *braziliensis* would be very useful for studies on the pathogenesis of the disease as well as a tool to develop new control strategies, such as vaccines and drugs.

The treatment of leishmaniasis relies on a few drugs, most of them displaying some inadequacy to present requirements. They are expensive, toxic and require prolonged and parenteral administration. Additionally, drug resistance has been reported [11, 12]. More than a decade ago, miltefosine (MF) was described as an oral drug against visceral leishmaniasis (VL) and has been widely used in the Indian subcontinent for that application [13–15]. On the other hand, MF efficacy for the treatment of cutaneous leishmaniasis (CL) seems to be dependent on the *Leishmania* species and even on intraspecies heterogeneity. Some clinical studies in South America identified cure rates varying from 53% to 91% in CL patients [16–18]. Two clinical studies performed in Brazil detected cure rates of 71.4% and 75%, respectively, in infections due to *L*. *guyanensis* [19] and *L*. *braziliensis* [20]. However, given the lack of other new treatment alternatives, further understanding of MF’s potentialities and limitations is necessary.

We have recently described the use of luciferase as a reporter for drug efficacy evaluation in cutaneous and visceral leishmaniasis models [21, 22]. In both models, luciferase has proven to be an accurate measure of parasite burden.

In this study, we generated a transgenic line of *L*. *braziliensis* expressing luciferase that was adapted to induce chronic lesions in BALB/c mice. Using this experimental model, we evaluated the efficacy of MF to reduce lesion size and parasite load.

## Methods

### Drugs

Amphotericin B sodium deoxycholate and MF were obtained from Sigma-Aldrich (St. Louis, MO, USA). Stock solutions of amphotericin B and MF for *in vitro* experiments were prepared in sterile water (10 mM). Miltefosine used for the treatment of infected mice was prepared daily in sterile saline (0.9% NaCl) from a 6 mg/mL stock solution.

### Parasites

The *L*. *braziliensis* strain MHOM/BR/94/H3227 was kindly provided by Dr. Maria Jania Teixeira, from Universidade Federal do Ceará. These parasites were isolated from a cutaneous ulcer from a patient in Ceará State, Brazil and the strain was previously typed as *L*. *braziliensis* [10]. Promastigotes were grown in medium M199 (Sigma-Aldrich) supplemented with 10% heat-inactivated foetal calf serum, 2% urine, 0.25% hemin, 12 mM NaHCO_3_, 50 U/mL penicillin and 50 μg/mL streptomycin at 25°C. For *in vivo* experiments, female BALB/c mice (4–5 week-old) were inoculated with 10^6^ stationary-phase promastigotes injected subcutaneously in the right hind footpad. Amastigotes were obtained from infected mice, as described [23] and differentiated back as promastigotes in M199. Promastigotes were counted in a Neubauer haemocytometer.

### Generation of a transgenic line of *L*. *braziliensis* expressing luciferase

**Fig 1:**
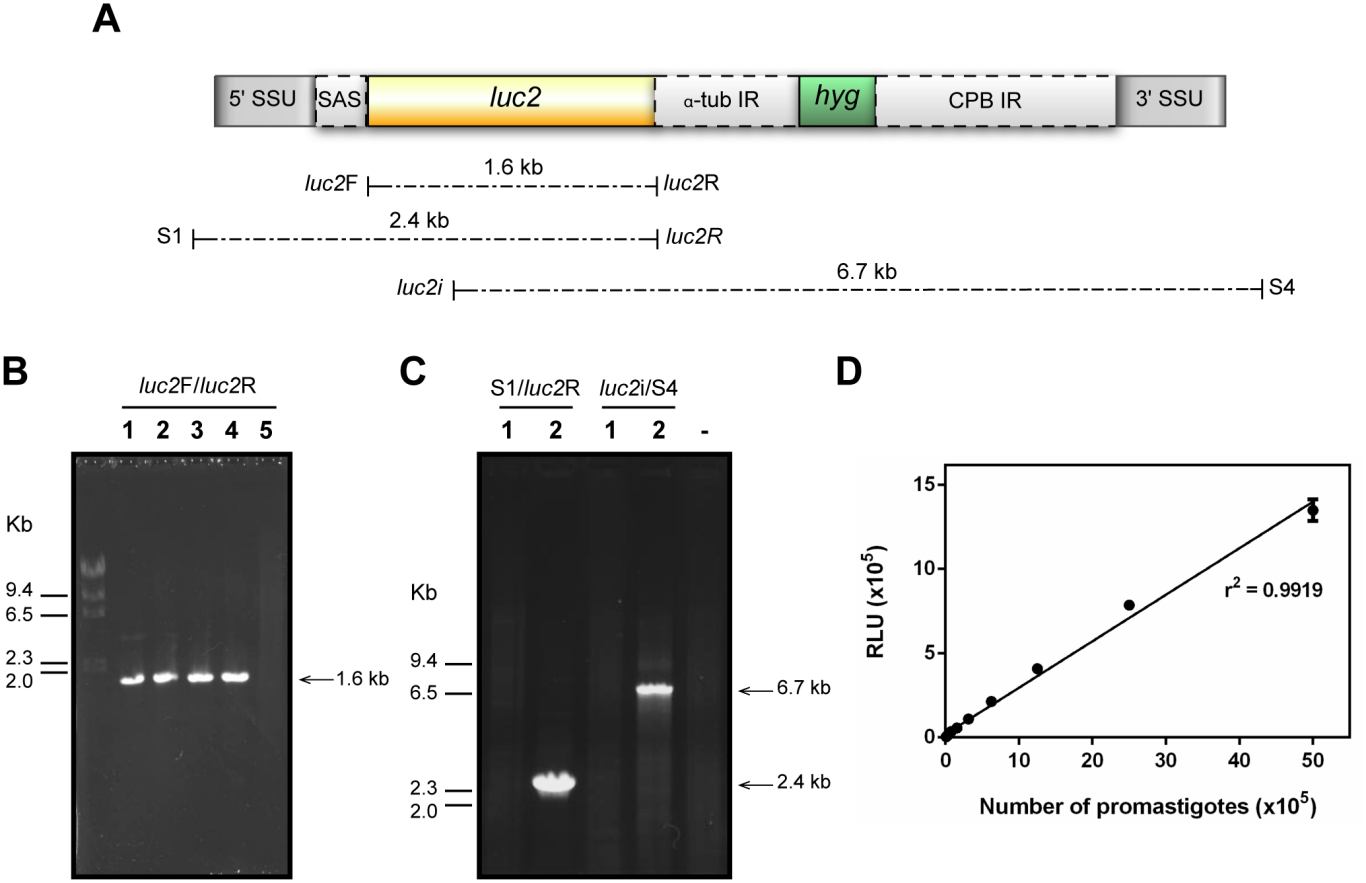
Characterisation of a transgenic line of *L*. *braziliensis* expressing luciferase. (A) Schematic representation of the linear cassette for integration in the SSU rDNA *locus*. SSU, small subunit; SAS, splice acceptor site; *luc2*, *luc2* coding sequence; α-tub IR, intergenic region of the *L*. *enrietii* α-tubulin gene; *hyg*, hygromycin phosphotransferase gene; CPB IR, *L*. *mexicana* cysteine protease B gene intergenic region. Position of the primers and size of amplified fragments are indicated by the dotted lines. (B) Size separation of PCR products amplified using primers complementary to the *luc2* ORF extremities from genomic DNA purified from four independent transfected clones (lanes 1 to 4) and from the wild type strain (lane 5). (C) Agarose gel electrophoresis of amplified products obtained from *Lb*-WT (lane 1) or *Lb*-LUC (lane 2) with the pairs of primers S1/luc2*R* or luc2*i*/S4. (-) indicates the control reaction without template DNA. (D) Luminescence intensity and number of *Lb*-LUC promastigotes. Promastigotes were serially diluted and luminescence was measured using a microplate reader, after addition of luciferin. RLU, relative light units.

A construct containing the *luc2* gene was previously built [22] taking advantage of the vectors pSSU-int and pSPαHYGα which were kindly provided by Dr. Tony Aebischer (Robert Koch Institute, Berlin, Germany) and Dr. Marc Ouellette (Universite Laval, Quebec, Canada). The linear cassette purified upon *Pac* I and *Pme* I digestion of the construct described previously, was used to transfect *L*. *braziliensis* H3227 promastigotes. Briefly, the cassette contains the *luc2* gene followed by a *Leishmania* 3’ UTR, the hygromycin phosphotransferase gene and fragments of the *L*. *mexicana* small subunit (SSU) ribosomal DNA (rDNA) at the cassette extremities to promote homologous recombination. Transfection was performed as described [24] using 5 μg of linear DNA. Twenty-four hours after transfection, 32 μg/mL hygromycin B was added for selection of mutants. The *L*. *braziliensis* transfectant line was kept for three passages in M199 containing hygromycin B and then plated on semi-solid M199 medium/1% agar supplemented with 1.2 μg/mL biopterin, 2% urine and 32 μg/mL hygromycin B for cloning [24]. Four independent clones were analysed for integration of the cassette into the rDNA *locus* by PCR amplification, using primers complementary to sequences inside and outside of the linear cassette. Genomic DNA of these clones was purified as described [25] for confirmation by PCR. Primers used were S1 (5’-GATCTGGTTGATTCTGCCAG-3’) and S4 (5’-GATCCAGCTGCAGGTTCACC-3’) [26] that anneal to the SSU rDNA sequence flanking the insertion sites and primers luc2*F* (5’-GCGGGATCCATGGAAGATGCCAAAAACATTAAG-3’), luc2*R* (5’-CACGCGCATACATTCACGGCGTTACACGGCGATCTTGCCGC-3’) and luc2*i* (5’-GACCGACTACCAGGGCTTCC-3’) that anneal to the *luc2* gene contained in the linear cassette (Fig 1A) [22].

### Parasite susceptibility to drugs *in vitro*

Susceptibility of *L*. *braziliensis* wild type and transgenic lines to amphotericin B and MF was evaluated by a [3-(4,5-dimethylthiazol-2-yl)-2,5-diphenyl tetrazolium bromide] (MTT, Sigma-Aldrich) viability test assay as described [27]. For this assay, promastigotes (2×10^6^ parasites per well) were incubated in the presence of increasing concentrations of amphotericin B (0.037 to 0.3 μM) or MF (25 to 200 μM) for 24 h. MTT cleavage was measured in a microplate reader (POLARstar Omega, BMG Labtech, Ortenberg, Germany) with a test wavelength of 595 nm and a reference wavelength of 690 nm.

For intracellular amastigotes, bone marrow-derived macrophages (BMDM) from BALB/c mice were used as previously described [21]. BMDM were plated on round glass coverslips in 24-well culture dishes, at a density of 3 × 10^5^ cells in 400 μL of RPMI 1640 medium (Gibco, Invitrogen Corporation) supplemented with 10% FCS (Gibco, Invitrogen Corporation) in a 5% CO_2_ atmosphere for 16 h at 37°C allowing macrophages to adhere. Macrophages were then infected with stationary-phase promastigotes using ratios varying from 20 to 35 parasites per macrophage for 3 h at 33°C. Non-internalized parasites were removed by washing with warmed PBS, followed by the addition of fresh medium containing increasing concentrations of amphotericin B (0.025 to 0.2 μM) or MF (0.5 to 24 μM). After 48 h, the cells were fixed in methanol and stained using the panoptic haematological labelling method (Instant Prov kit, Newprov, Pinhais, PR, Brazil). The percentage of infected macrophages was determined by counting 100 cells in three independent experiments. The half-maximal effective concentrations (EC_50_) were determined from sigmoidal regression of the concentration-response curves. Assays were performed in triplicate and results are expressed as the mean and standard deviation (SD) of at least three independent experiments.

### Luciferase assay *in vitro*

The activity of luciferase was measured in recombinant *L*. *braziliensis* promastigotes at logarithmic and stationary growth phases. Parasites were washed twice with PBS and then serially diluted for the luciferase assay, which was performed using the One Glo Luciferase Assay System (Promega Corporation) according to the manufacturer’s instructions and as described previously [22]. Bioluminescence was measured in a microplate reader (POLARstar Omega, BMG Labtech, Ortenberg, Germany). Each point was tested in triplicate in two independent experiments.

### Animal infections

Male golden hamsters (3 to 5 weeks-old) were obtained from the Instituto de Medicina Tropical, University of São Paulo. Female BALB/c mice (3 to 5 weeks-old) were from the Instituto de Ciências Biomédicas, University of São Paulo. Animals were kept in cages and received unlimited food and water.

Given that the wild type (*Lb*-WT) and luciferase transgenic parasites (*Lb*-LUC) were in different passages as promastigote cultures, they were initially used to inoculate highly susceptible hamsters. Initially, 10^6^ stationary phase promastigotes were injected into hamsters’ hind footpads in a volume of 30 μL. Once the infections were established, parasites of both lines were recovered from the inoculated footpads and differentiated back into promastigotes in M199 at 25°C. These promastigotes were then used to inoculate BALB/c mice using the same protocol described above, except for the host. When the lesions developed, parasites were recovered, transformed into promastigotes and used to inoculate mice and hamsters with the results described in the Results section. Disease progression was followed for 11 weeks in hamsters and for 24 weeks in BALB/c mice.

### Treatment of mice with MF and evaluation of treatment efficacy

BALB/c mice inoculated with *Lb*-WT or *Lb*-LUC were submitted to treatment with MF. Treatment was initiated 4 weeks post-inoculation, using two different doses of the drug: 5 or 15 mg/kg/day, in groups of 5 animals. MF was prepared daily from a stock solution of 6 mg/mL and was administered by oral gavage for 15 consecutive days. An untreated group was used as a control for each line studied. Lesion sizes were evaluated once a week by measuring the difference in thickness between the infected and the contralateral uninfected footpad using a caliper (Mitutoyo Corporation, Kawasaki, Kanagawa, Japan).

Parasite burden in *Lb*-LUC inoculated BALB/c mice was determined 6 weeks (at the end of MF treatment), 17 and 23 weeks post-inoculation. The parasite load was quantified by measuring luciferase activity through bioimaging (IVIS Spectrum, Caliper Life Sciences, Inc. MA/USA) as described [21]. Briefly, before luminescence detection animals received 75 mg/kg luciferin (VivoGlo Luciferin, Promega) intraperitoneally followed by anesthesia in a 2% isoflurane atmosphere (Cristália). Images were collected after 20 minutes using a high-resolution mode with 2 minutes exposure from a fixed-size region of interest. Results were quantified with Living Image software version 4.3.1 (Caliper Life Sciences) and were expressed as photons/second/square centimeter/steradian (ph/sec/cm^2^/sr).

### Susceptibility to MF after *in vivo* treatment

*Lb*-WT parasites were recovered from infected mice that had been treated with 5 mg/kg/day (n = 5) or with 15 mg/kg/day (n = 5) MF at the 19^th^ and 23^th^ week post-inoculation, respectively. These parasites were differentiated into promastigotes in M199 medium and their susceptibility to MF was determined by the MTT assay as described above. As controls, parasites were also rescued from an untreated infected animal at the 19^th^ week post-infection.

### Statistical analysis

Unpaired two-tailed Student’s *t* tests were used to compare the EC_50_ determined *in vitro* on promastigotes and intracellular amastigotes of wild type parasites and luciferase expressing lines and for comparing disease progression. Parasite burden was analysed for statistical significance by One Way ANOVA, followed by the Tukey post-test. All statistical analyses were performed using GraphPad Prism 6.0 software (GraphPad Software, Inc., La Jolla, CA USA). Results were considered significant at P < 0.05.

### Ethics statement

Animal experiments were approved by the Ethics Committee for Animal Experimentation of the Biomedical Sciences Institute (Protocol: 178/138/02) and of the Tropical Medicine Institute (Protocol: CPE-IMT 2012/145) of the University of São Paulo in agreement with the guidelines of the Sociedade Brasileira de Ciência de Animais de Laboratório (SBCAL) and of the Conselho Nacional de Controle da Experimentação Animal (CONCEA).

## Results

### Generation and characterisation of a luciferase expressing line of *L*. *braziliensis*

To generate a luciferase expressing *L*. *braziliensis* line (*Lb*-LUC), promastigotes of the H3227 strain were transfected with a linear cassette containing the *luc2* gene flanked by SSU rDNA sequences (Fig 1A). After transfection, parasites were selected in the presence of hygromycin B and cloned. Four independent clones were tested for the presence of the *luc2* gene by PCR. Amplification of a 1.6 kb fragment with oligonucleotides complementary to the 5’ and 3’ ends of the *luc2* open reading frame was observed in the four selected clones while the same reaction led to the absence of amplification from wild type parasites (Fig 1B). Additional pairs of primers were used to confirm the correct integration into the ribosomal DNA *locus* in one of the selected clones (clone 3, lane 3 in Fig 1B). Primers S1 and S4 [26], complementary to the rDNA outside the linear cassette, were used in combination with primers luc2*R* and luc2*i*, respectively (Fig 1A). These two combinations of primers amplified the expected fragments of 2.4 and 6.7 kb in the transgenic line but not in wild type parasites (Fig 1A and 1C).

Bioluminescence in the transgenic line (*Lb*-LUC) was confirmed after incubation of parasites with luciferin. A linear correlation between the number of promastigotes and the production of light was observed (Fig 1D).

Growth curves for transgenic *Lb*-LUC and wild type (*Lb*-WT) promastigotes were indistinguishable (S1A Fig). Logarithmic and stationary phase promastigotes presented similar light emission (S1B Fig) indicating that luciferase expression was stable along the growth curve. The stability of the transgene was also evaluated by removing the drug pressure from promastigote cultures. Luciferase expression was stable with the same levels of light production in parasites kept in the absence of hygromycin B for 25 passages in culture (S1C Fig).

No differences were observed in the infectivity of *Lb*-LUC and *Lb*-WT to macrophages after 24 or 48 hours evaluated by the morphology of infected cells, percentage of infected cells or number of amastigotes per infected macrophage (S2A–S2D Fig). In addition, a direct correlation was observed between the number of *Lb*-LUC intracellular amastigotes per macrophage and bioluminescence (S2E Fig). Finally, *in vitro* susceptibility of *Lb*-LUC and *Lb*-WT promastigotes and intracellular amastigotes to amphotericin B and MF showed no significant differences (Table 1).

**Table 1:** Drug susceptibility in promastigotes and intracellular amastigotes of the wild type and the transgenic line of *L*. *braziliensis* H3227.

Drug	EC_50_ ± S.D. ^a^			
	Promastigotes		Intracellular amastigotes	
	*Lb*-WT	*Lb*-LUC	*Lb*-WT	*Lb*-LUC
amphotericin B	0.11 ± 0.01	0.11 ± 0.02	0.05 ± 0.02	0.05 ± 0.01
miltefosine	40.70 ± 8.55	47.71 ± 5.85	0.99 ± 0.05	0.91 ± 0.01

^a^ Half maximal effective concentration ± standard deviation (in μM). Results are the average of at least three independent experiments.

### Disease progression in hamsters and BALB/c mice inoculated with *Lb-*LUC

**Fig 2:**
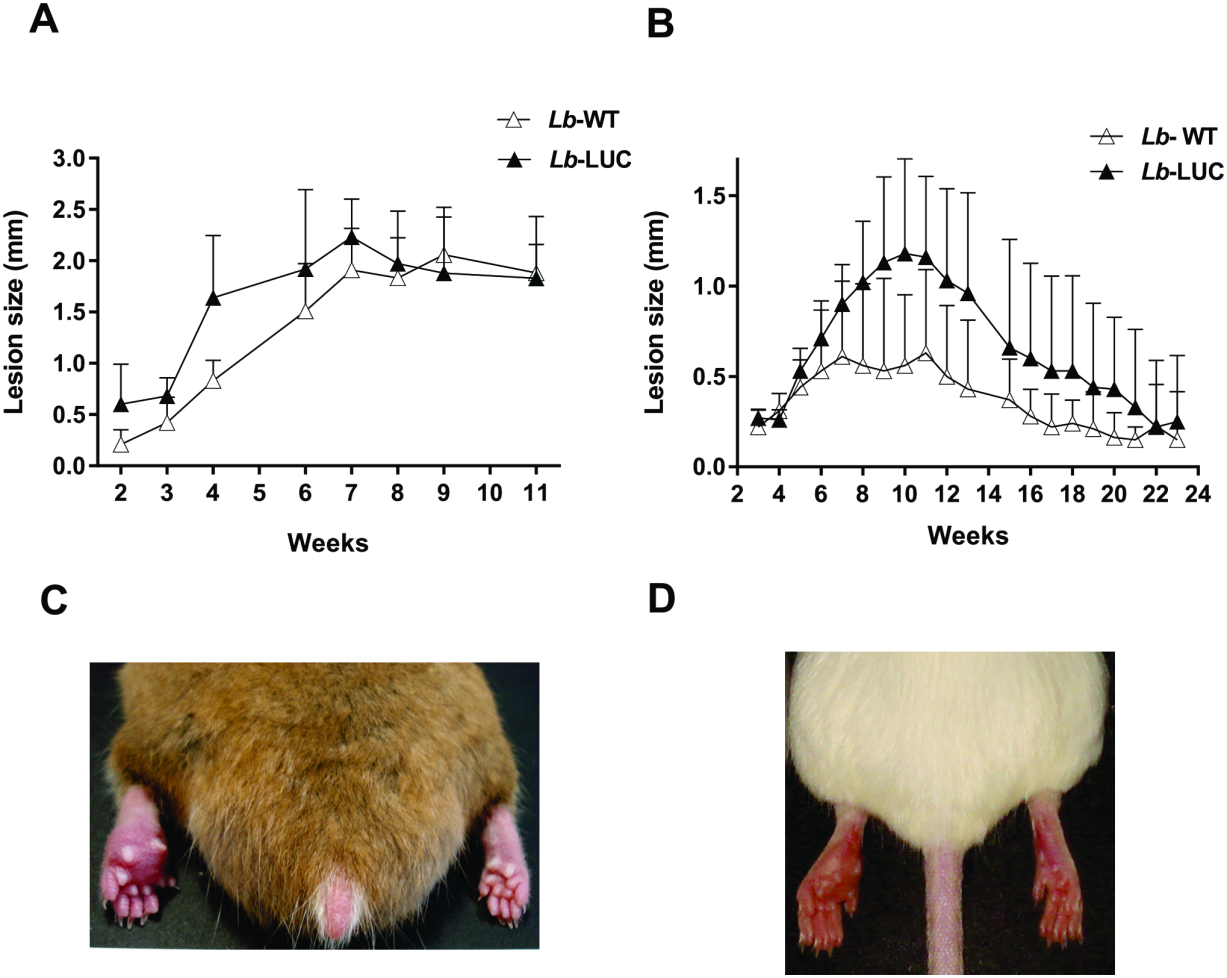
Course of disease after *L*. *braziliensis* H3227 inoculation in hamsters and BALB/c mice. Evolution of lesion size in hamsters (A) and BALB/c mice (B) inoculated with *Lb*-LUC or *Lb*-WT H3227 strain. Animals were inoculated with 10^6^ stationary-phase promastigotes in the left hind footpad and lesion size was measured weekly (five animals per group). Data is the mean and standard deviation from one experiment representative of two independent experiments. Student’s *t* test comparing the area under the curve detected non-statistically significant differences with *P* = 0.25 (A) and *P* = 0.11 (B). Representative images of a hamster at the 5^th^ week (C) and of a BALB/c mouse at the 6^th^ week post-inoculation (D) with H3227 *Lb*-LUC parasites. The left footpad (inoculated) and right footpad (control) of a hamster (C) and a BALB/c mouse (D), representative of each group of animals, are shown.

Hamsters and BALB/c mice were inoculated with 10^6^ *Lb*-WT or *Lb*-LUC promastigotes subcutaneously in the footpad. In both animal models, there were no significant differences in the kinetics of lesion development or in lesion size when the two parasite lines were compared (Fig 2A and 2B). In both models, lesions were non-ulcerative (Fig 2C and 2D). In hamsters, lesions were detectable from two weeks post-inoculation, progressed steadily for the next five weeks and then the oedema stabilized till the 11^th^ week post-inoculation (Fig 2A), when animals were euthanized. In BALB/c mice, lesions were clearly detected after two weeks, progressed in size until weeks 9–10 and then decreased in the following weeks (Fig 2B and S3 Fig), but the local oedema remained detectable until 23 weeks post-inoculation (Fig 2B and S3 Fig). Therefore, no spontaneous healing was observed in the animal models studied during the period evaluated.

**Fig 3:**
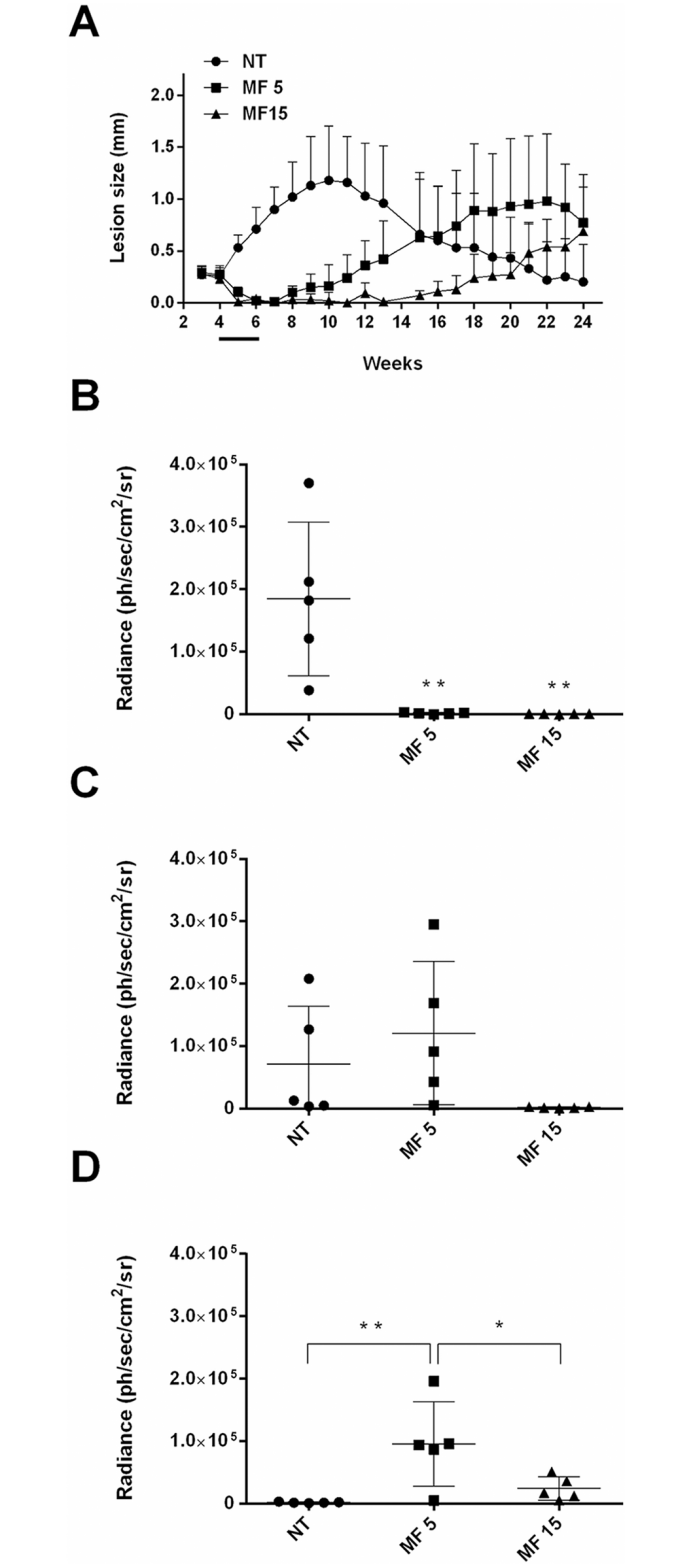
*In vivo* efficacy of miltefosine in a *L*. *braziliensis*-inoculated BALB/c mice model. 10^6^ *Lb*-LUC stationary promastigotes were injected in the left hind footpad and animals were treated with 5 or 15 mg/kg/day of miltefosine for 15 consecutive days (from to 4^th^ to the 6^th^ week post-inoculation). Average lesion size was measured weekly (five animals per group) in untreated and treated animals and horizontal black bar indicates MF treatment (A). Parasite burden was evaluated by bioluminescence at the end of the treatment (6^th^ week post-inoculation) (B), at the 17^th^ (C) and at the 23^th^ week post-inoculation (D) (40, 120 and 160 days post-inoculation respectively). Statistical analysis was performed with One Way ANOVA, followed by the Tukey post-test. * *p* < 0.05; **, *p* < 0.001. Ph/sec/cm^2^/sr, photons per second per square centimetre per steradian.

**Fig 4:**
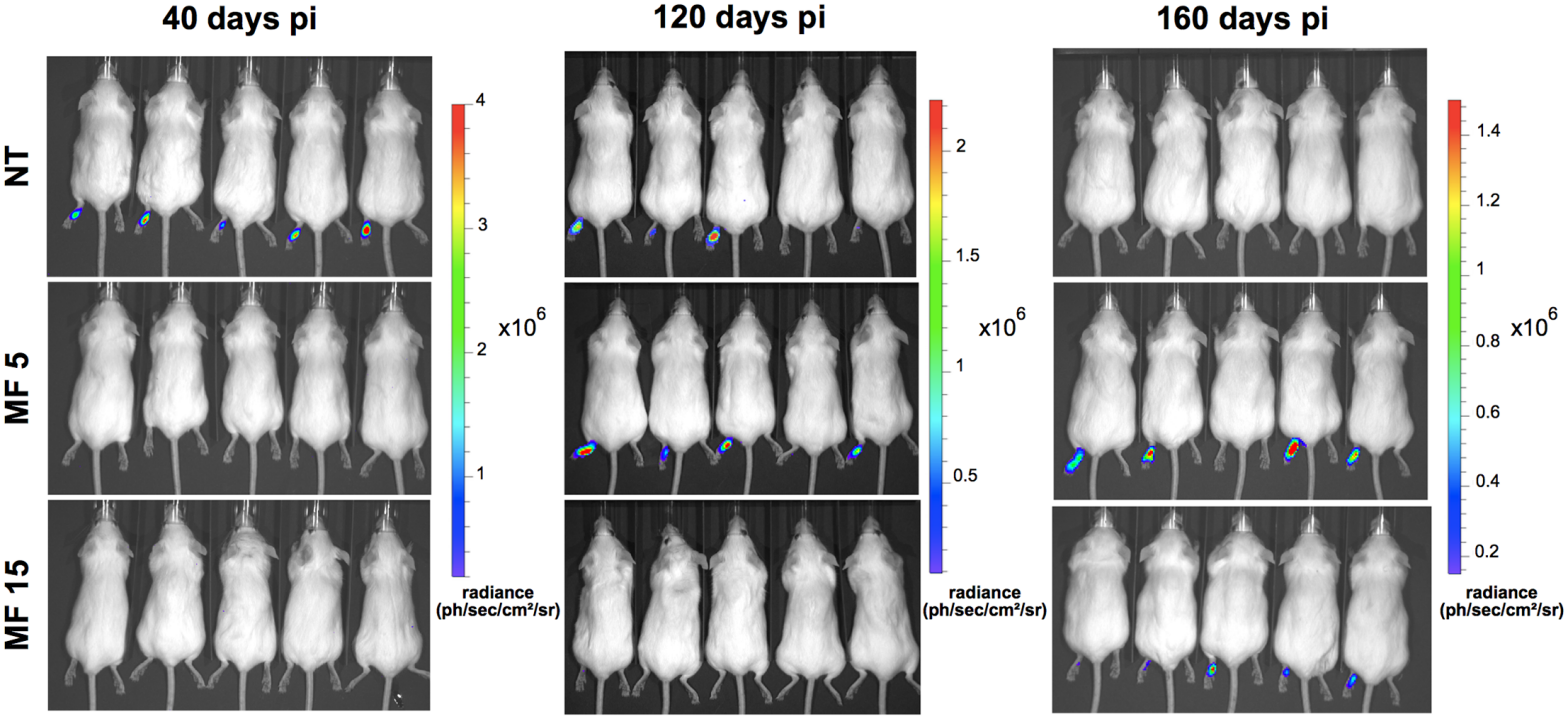
Parasite burden of left hind footpad of BALB/c mice treated with MF and evaluated by bioluminescence. Groups of 5 animals untreated (NT), or treated with 5 or 15 mg/kg/day MF (MF 5 and MF 15 respectively) were evaluated at 40, 120 and 160 days (6^th^, 17^th^ and 23^th^ week respectively) post-inoculation (pi). The bars on the right show a pseudo-colour scale representing light intensities.

BALB/c mice inoculated with *Lb*-LUC were examined by *in vivo* imaging after 6, 17 and 23 weeks (Figs 3 and 4). Luminescence was detected from the lesion site in all mice at the 6^th^ week post-inoculation (Figs 3B and 4). By week 17^th^, light emission was not detected in 2 out of 5 mice (Figs 3C and 4). No bioluminescence was detected in untreated animals at the 23^th^ week post-inoculation (Figs 3D and 4), although measurable oedema was still observed in this group at the 24^th^ week (Fig 3A).

### *In vivo* efficacy of MF in *Lb*-LUC inoculated BALB/c mice

The late-healing infection model of *L*. *braziliensis* H3227 in BALB/c mice was used to test MF efficacy. Treatment was initiated 4 weeks post-inoculation in animals injected with the transgenic line *Lb*-LUC (Fig 3) or with the wild type parasites (S3 Fig). Animals were treated with MF 5 or 15 mg/kg/day for 15 consecutive days. Both doses of MF resulted in regression of lesions at the end of the treatment (6 weeks post-inoculation) (Fig 3A and S4 Fig). Luminescence was not detected by bioimaging at the end of the treatment (Figs 3B and 4).

However, 8 weeks post-inoculation a relapse was observed in animals treated with 5 mg/kg/day MF, with lesions progressing in size for the following weeks (Fig 3A and S3 Fig). These findings were confirmed by quantification of bioluminescence 120 days post-inoculation (17^th^ week), time when animals treated with 5 mg/kg/day presented a higher parasite burden than untreated animals (Figs 3C and 4).

A clinically detectable relapse was also observed in animals treated with 15 mg/kg/day from the 15^th^ week post-inoculation that progressed in the following weeks (Fig 3A). Although no luminescence was detected in these animals at week 17^th^ (120 days) post-inoculation (Figs 3C and 4), parasites were clearly detected by *in vivo* imaging at the 23^th^ week post-inoculation (Figs 3D and 4).

Animals inoculated with *Lb*-WT and treated with both doses of MF presented the same pattern of disease progression, response and relapse to treatment observed with the *Lb*-LUC transgenic line (Fig 3A and S3 Fig).

To investigate whether these relapses were due to MF resistance acquired during the therapy regimen, parasites were recovered from 5 animals treated with MF 5 mg/kg/day (at the 19^th^ week post-inoculation) and with 15 mg/kg/day (at the 23^th^ week post-inoculation) (S3 Fig). After differentiation to promastigotes, MF susceptibility was determined in parasites isolated from untreated and treated animals and no significant changes in drug susceptibility were found between these parasites (Table 2).

**Table 2:** Susceptibility of promastigotes to MF after recovery from BALB/c mice treated with 5 or 15 mg/kg/day.

Treatment regimen	*Lb*-WT lines ^a^	EC_50_ ± S.D. ^b^
Untreated	*Lb*-WT (NT.1)	40.88 ± 6.77
5 mg/kg/day	*Lb*-WT (MF5.1)	36.82 ± 6.10
	*Lb*-WT (MF5.2)	34.33 ± 8.33
	*Lb*-WT (MF5.3)	38.12 ± 9.75
	*Lb*-WT (MF5.4)	41.45 ± 3.80
	*Lb*-WT (MF5.5)	43.26 ± 9.05
15 mg/kg/day	*Lb*-WT (MF15.1)	28.24 ± 2.31
	*Lb*-WT (MF15.2)	28.20 ± 4.46
	*Lb*-WT (MF15.3)	30.80 ± 4.76
	*Lb*-WT (MF15.4)	29.00 ± 9.78
	*Lb*-WT (MF15.5)	36.34 ± 8.68

^a^ Amastigotes were rescued from *Lb*-WT inoculated BALB/c mice treated with MF 5 mg/kg/day (5 animals), 15 mg/kg/day (5 animals) or untreated (one animal) (MF5, MF15 and NT.1 respectively), differentiated in M199 to promastigotes and then tested for MF susceptibility by MTT as described in Methods.

^b^ Half maximal effective concentration ± standard deviation (in μM). Results are the average of at least three independent experiments.

## Discussion

To the best of our knowledge, this is the first report of a luciferase-expressing *L*. *braziliensis*, the causative species of cutaneous and mucocutaneous leishmaniasis in humans and the most prevalent species in South America [2]. The importance of research on *L*. *braziliensis* infections cannot be overstated. However, it faces multiple difficulties largely derived from the distinct behaviour of this *Leishmania* species, *in vitro* and *in vivo*. *L*. *braziliensis* promastigotes do not grow easily in axenic cultures and amastigotes are scarce in the lesions. The lack of an appropriate animal model mimicking the human infection in all of its potential clinical presentations is perhaps one of the main drawbacks. Most *L*. *braziliensis* strains are infective to mice but lead to asymptomatic or short-lived, self-healing disease [28, 29]. The infection is more sustained in hamsters [6, 29], but do not progress to a mucosal or disseminated disease.

The development of lesions at the site of inoculation with the H3227 strain of *L*. *braziliensis* in BALB/c mice was previously described [10]. However, in the original description, lesions were self-limited and healed 30 days post-inoculation. This strain was kindly given to us by Dr. Maria Jania Teixeira (Universidade Federal do Ceará, Brazil) and, before being submitted to transfection, was passaged in mice repeatedly. Most likely as a result of these repeated *in vivo* passages, the line obtained after transfection, together with the parental wild type parasites were then able to induce a late-healing disease in BALB/c mice, as well as being pathogenic in hamsters. A late-healing animal model of *L*. *braziliensis* infection is likely to become very useful as a tool in drug development or in the evaluation of the disease pathogenesis.

It is unclear at present whether other strains of *L*. *braziliensis* could be adapted to cause late-healing disease in BALB/c mice. We have attempted to do so with three other isolates as well as with the type strain of *L*. *braziliensis* M2903 without success but cannot rule out that possibility. It will be interesting to investigate whether mutations on this *Leishmania* isolate (H3227) are present and could be correlated with the altered phenotype.

These parasites were transformed to express the *luciferase* gene integrated into the ribosomal *locus*. Luciferase expression was stable throughout the life cycle. As previously reported for other luciferase-expressing *Leishmania* species [21, 22, 30], this *L*. *braziliensis* transgenic line presented the same biological properties *in vitro* and *in vivo* of the parental line and was useful for parasite quantification of promastigotes and amastigotes, *in vitro* and *in vivo*. Luciferase as a reporter has become instrumental in pre-clinical drug development projects, especially in chronic infections. Our previous results with *L*. *amazonensis* [21] and *L*. *chagasi* [22] lines expressing luciferase clearly demonstrated that luciferase detection correlates well with parasite burden *in vivo*. In the case of H3227 parasites, the sensitivity of detection *in vivo* has proven to be insufficient to detect low parasite burdens, as was the case in *Lb*-LUC in untreated animals or when relapses were noticed in the groups treated with the higher dose of MF. This limitation in sensitivity should be taken into account in drug efficacy studies. Nevertheless, bioimaging was used successfully to document infection and as a semi-quantitative method for comparing parasite burden between different groups.

The model of infection with *Lb*-LUC in BALB/c mice was put to test using MF, the newest addition to leishmaniasis chemotherapy. The susceptibility of the H3227 strain to MF *in vitro* can be considered comparable to other *L*. *braziliensis* strains [31, 32]. Accordingly, at the end of MF treatment, *Lb*-LUC inoculated mice showed regression of lesions and absence of luminescence. However, although the doses used here were higher than the WHO recommended scheme for CL and VL (2.5 mg/kg/day) [15], a clinical relapse was evident in animals treated with 5 mg/kg/day as soon as two weeks after the end of treatment. In these animals, lesions progressed in the following weeks reaching sizes similar to the ones observed in untreated animals. In animals treated with 15 mg/kg/day, oedema in the footpad reappeared 10 weeks after the interruption of treatment.

Relapses in VL patients treated with MF do not seem to be due to acquired resistance [33]. Likewise, our data showed similar MF susceptibility in parasites rescued from untreated and treated animals, indicating that *L*. *braziliensis* did not acquire resistance to MF during treatment.

In VL patients, MF failure may be correlated with low drug exposure due to pharmacokinetic-pharmacodynamics factors [34]. MF has been shown to reach high levels in the skin [15] but poor drug distribution to cutaneous lesions may be another reason for the lower efficacy of the drug against CL when compared with VL. Furthermore, the greater genetic diversity of *L*. *braziliensis* compared to *L*. *donovani* [35, 36] may increase the number of elements that contribute to treatment failure. Moreover, other factors, unrelated to drug resistance, should be considered as quiescence and even the presence of the LRV1 virus [37, 38].

The persistence of viable parasites after treatment with 5 and 15 mg/kg/day MF per 15 days indicated that, even for the highest dose used, clinical response did not reflect sterile cure. In fact, sterile cure does not seem to be required for long-term control of the disease, as has been shown by the presence of parasites in healthy skin in animals [39, 40]. On the other hand, relapses have been reported in patients treated with MF for all clinical forms of the disease [33, 41–46]. Therefore, we believe the data presented herein stress the need for long-term follow up of MF treated patients and suggest that higher doses and/or longer treatment regimens should be evaluated in order to avoid relapses. In addition, the combined use of MF with other antileishmanial drugs should be considered as an alternative for the chemotherapy of the disease.

## Supporting Information

S1 Fig Characterization of the transgenic line of *L*. *braziliensis* expressing luciferase. (A) Growth curves for *Lb*-WT and *Lb*-LUC promastigote lines. Parasites were grown in M199 and then counted using a Neubauer hemocytometer. Data is the mean and standard error from two independent experiments. RLU, relative luminescence units. (B) Luciferase activity in logarithmic and stationary-phase *Lb*-LUC promastigotes. (C) Stability of luciferase expression in *Lb*-LUC. Promastigotes were cultivated for 25 passages in the presence or absence of hygromycin B and then tested for luminescence.
(DOCX)

S2 Fig *In vitro* infectivity of *Lb*-LUC in BMDM. Macrophages were infected with stationary phase promastigotes for 3 hours at 33°C. Non-internalized parasites were removed by washing with warmed PBS. (A) and (B): Representative images of macrophages infected with *Lb*-WT (A) and *Lb*-LUC (B). (C) Percentage of infected macrophages after 24 or 48 hours. Cells were infected with a ratio of 20 parasites per macrophage. The percentage of infection was determined by counting 100 cells in triplicates. (D) Number of amastigotes per infected macrophage after 24 or 48 hours. (E) Macrophage infection evaluated through luminescence. Relative luminescence units (RLU) were determined for macrophages after infection with different ratios of parasites per macrophage (25:1, 30:1 and 35:1). The average and standard deviation of three independent experiments in triplicates is shown for both experiments. Anova with Tukey’s post test: (*) *P*< 0.05; (**) *P* <0.01; (***) *P* <0.001.
(DOCX)

S3 Fig *In vivo* efficacy of miltefosine in *L*. *braziliensis*-inoculated BALB/c mice. 10^6^ *Lb*-WT stationary-phase promastigotes were injected in the mice left hind footpad. Treatment with 5 or 15 mg/kg/day of miltefosine was given for 15 consecutive days (from 4^th^ to 6^th^ week post-inoculation). Average of lesion size was measured weekly (five animals per group) in untreated and treated animals The horizontal black bar indicates MF treatment and the arrows indicate the time points when parasites were recovered from BALB/c mice treated with 5 or 15 mg/kg/day (19^th^ and 23^th^ week respectively).
(DOCX)
