# Supplementary material for: A Luciferase-Expressing Leishmania braziliensis Line That Leads to Sustained Skin Lesions in BALB/c Mice and Allows Monitoring of Miltefosine Treatment Outcome
Source: PLoS Negl Trop Dis. 2016 May 4;10(5):e0004660. doi: 10.1371/journal.pntd.0004660 (PMC4856402; doi:10.1371/journal.pntd.0004660)
Supplement: S1 Fig — (A) Growth curves for Lb-WT and Lb-LUC promastigote lines. Parasites were grown in M199 and then counted using a Neubauer hemocytometer. Data is the mean and standard error from two independent experiments. RLU, relative luminescence units. (B) Luciferase activity in logarithmic and stationary-phase Lb-LUC promastigotes. (C) Stability of luciferase expression in Lb-LUC. Promastigotes were cultivated for 25 passages in the presence or absence of hygromycin B and then tested for luminescence. (DOCX) [file pntd.0004660.s001.docx]

**Coelho et al. - S1 Figure**

**

**

**Fig S1.** **Characterization of the transgenic line of *L. braziliensis* expressing luciferase.** (A) Growth curves for *Lb*-WT and *Lb*-LUC promastigote lines. Parasites were grown in M199 and then counted using a Neubauer hemocytometer. Data is the mean and standard error from two independent experiments. RLU, relative luminescence units. (B) Luciferase activity in logarithmic and stationary-phase *Lb*-LUC promastigotes. (C) Stability of luciferase expression in *Lb*-LUC. Promastigotes were cultivated for 25 passages in the presence or absence of hygromycin B and then tested for luminescence.
