# Supplementary material for: A Luciferase-Expressing Leishmania braziliensis Line That Leads to Sustained Skin Lesions in BALB/c Mice and Allows Monitoring of Miltefosine Treatment Outcome
Source: PLoS Negl Trop Dis. 2016 May 4;10(5):e0004660. doi: 10.1371/journal.pntd.0004660 (PMC4856402; doi:10.1371/journal.pntd.0004660)
Supplement: S2 Fig — Macrophages were infected with stationary phase promastigotes for 3 hours at 33°C. Non-internalized parasites were removed by washing with warmed PBS. (A) and (B): Representative images of macrophages infected with Lb-WT (A) and Lb-LUC (B). (C) Percentage of infected macrophages after 24 or 48 hours. Cells were infected with a ratio of 20 parasites per macrophage. The percentage of infection was determined by counting 100 cells in triplicates. (D) Number of amastigotes per infected macrophage after 24 or 48 hours. (E) Macrophage infection evaluated through luminescence. Relative luminescence units (RLU) were determined for macrophages after infection with different ratios of parasites per macrophage (25:1, 30:1 and 35:1). The average and standard deviation of three independent experiments in triplicates is shown for both experiments. Anova with Tukey’s post test: (*) P< 0.05; (**) P <0.01; (***) P <0.001. (DOCX) [file pntd.0004660.s002.docx]

**Coelho et al. – S2 Fig.**


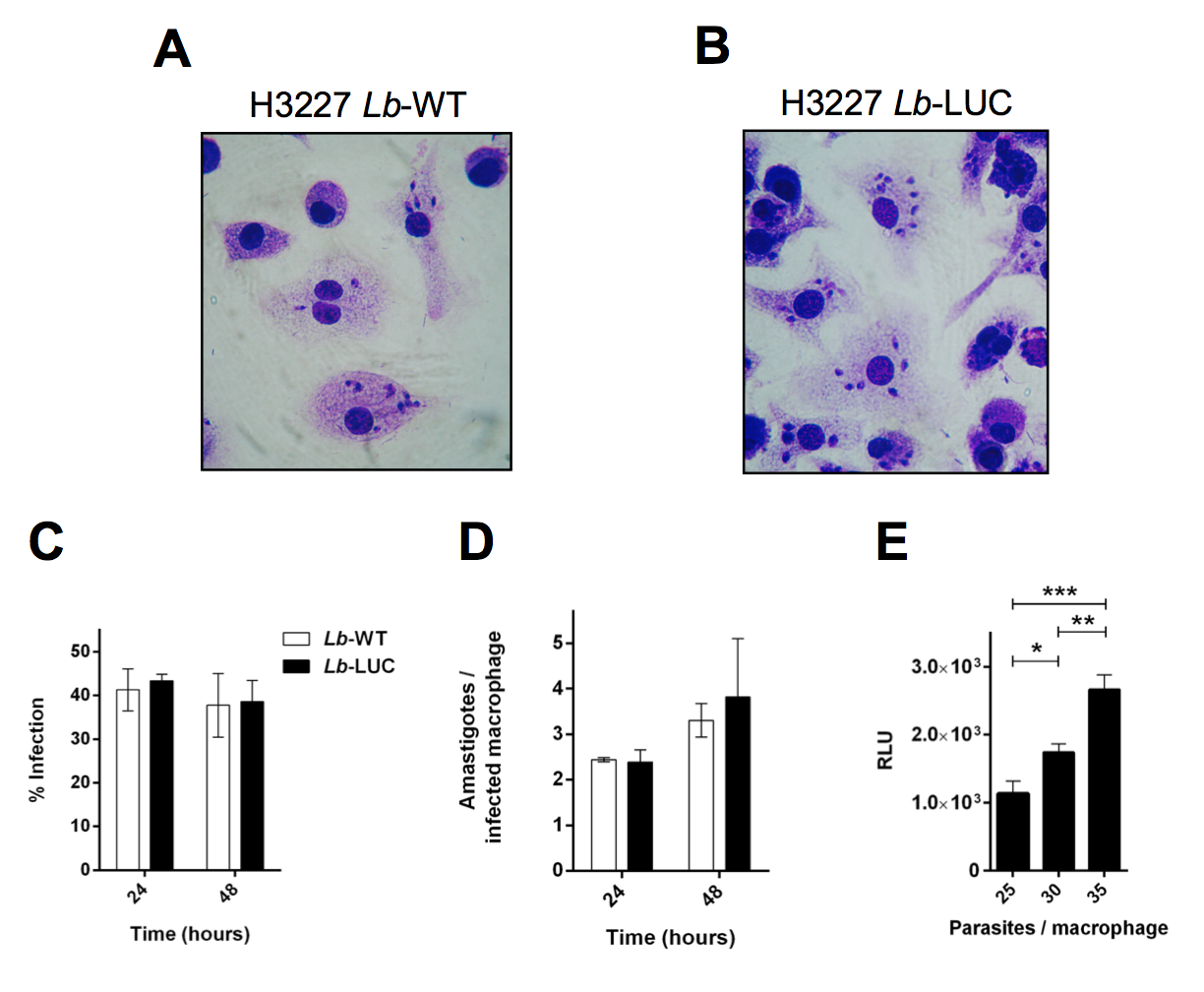


**Fig S2.** ***In vitro* infectivity of *Lb*-LUC in BMDM.** Macrophages were infected with stationary phase promastigotes for 3 hours at 33^o^C. Non-internalized parasites were removed by washing with warmed PBS. (A) and (B) Representative images of macrophages infected with *Lb*-WT (A) and *Lb*-LUC (B). (C) Percentage of infected macrophages after 24 or 48 hours. Cells were infected with a ratio of 20 parasites: macrophage. The percentage of infection was determined by counting 100 cells in triplicates. (D) Number of amastigotes per infected macrophage after 24 or 48 hours. (E) Macrophage infection evaluated through luminescence. Relative luminescence units (RLU) were determined for macrophages after infection with different ratios of parasites per macrophage (25:1, 30:1 and 35:1). The average and standard deviation of three independent experiments in triplicates is shown for both experiments. Anova with Tukey’s post test: (*) *P*< 0.05; (**) *P* <0.01; (***) *P* <0.001.
