# Supplementary material for: A Luciferase-Expressing Leishmania braziliensis Line That Leads to Sustained Skin Lesions in BALB/c Mice and Allows Monitoring of Miltefosine Treatment Outcome
Source: PLoS Negl Trop Dis. 2016 May 4;10(5):e0004660. doi: 10.1371/journal.pntd.0004660 (PMC4856402; doi:10.1371/journal.pntd.0004660)
Supplement: S3 Fig — 106 Lb-WT stationary-phase promastigotes were injected in the mice left hind footpad. Treatment with 5 or 15 mg/kg/day of miltefosine was given for 15 consecutive days (from 4th to 6th week post-inoculation). Average of lesion size was measured weekly (five animals per group) in untreated and treated animals The horizontal black bar indicates MF treatment and the arrows indicate the time points when parasites were recovered from BALB/c mice treated with 5 or 15 mg/kg/day (19th and 23th week respectively). (DOCX) [file pntd.0004660.s003.docx]

**Coelho et al. S3 Fig.**

**
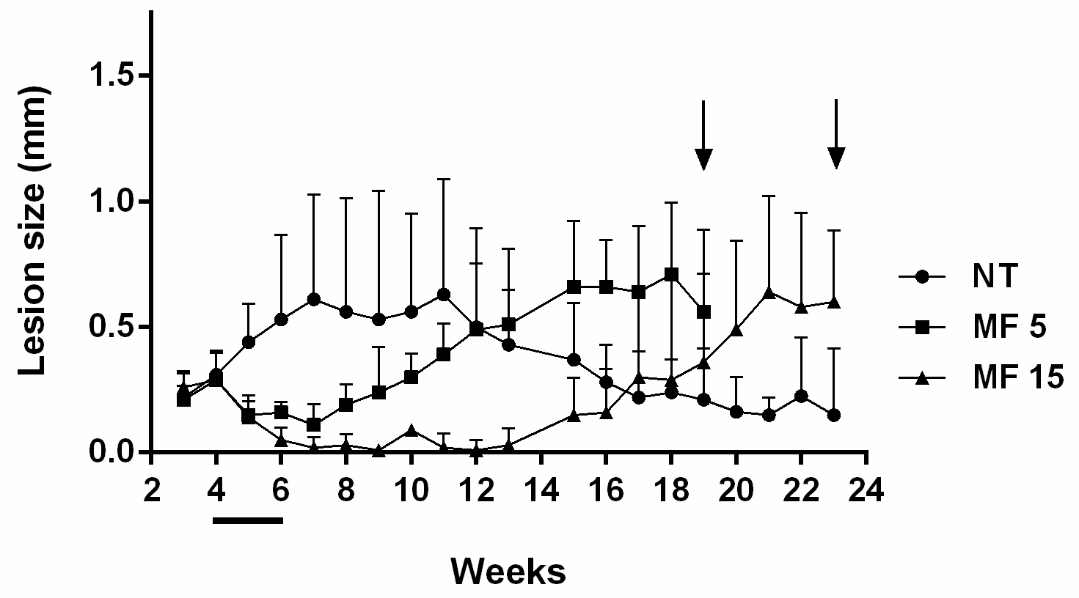
**

**S3 Fig.** ***In vivo* efficacy of miltefosine in *L. braziliensis*-inoculated BALB/c mice.** 10^6^ *Lb*-WT stationary-phase promastigotes were injected in the mice left hind footpad. Treatment with 5 or 15 mg/kg/day of miltefosine was given for 15 consecutive days (from 4^th^ to 6^th^ week post-inoculation). Average of lesion size was measured weekly (five animals per group) in untreated and treated animals The horizontal black bar indicates MF treatment and the arrows indicate the time points when parasites were recovered from BALB/c mice treated with 5 or 15 mg/kg/day (19^th^ and 23^th^ week respectively).
